# Neural circuits mediating food cue-reactivity: Toward a new model shaping the interplay of internal and external factors

**DOI:** 10.3389/fnut.2022.954523

**Published:** 2022-10-05

**Authors:** Francantonio Devoto, Carol Coricelli, Eraldo Paulesu, Laura Zapparoli

**Affiliations:** ^1^Psychology Department and NeuroMi—Milan Centre for Neuroscience, University of Milano-Bicocca, Milan, Italy; ^2^Psychology Department, Western University, London, ON, Canada; ^3^fMRI Unit, Istituto di Ricovero e Cura a Carattere Scientifico (IRCCS) Galeazzi, Milan, Italy

**Keywords:** food, cue-reactivity, neuroimaging, food craving, obesity

## Introduction

Growing evidence suggests that food and drug cues may activate similar brain networks (1), pointing to shared brain abnormalities in obesity and substance use disorder. These are of crucial importance, given the worldwide cost in individuals' health, wellbeing, and direct and indirect health costs (2, 3). Neuroimaging research has focused on the cue-reactivity paradigm (4), an experimental procedure involving the examination of neurofunctional responses to the controlled exposure to stimuli (food- or drug-related) inducing craving, namely the strong and intense desire to seek and consume a substance. These studies have revealed a distributed network of brain regions recruited during exogenous (i.e., perceptual food pictures, odors, or tastes) and endogenous (i.e., imagery) cue-reactivity [see (5) for a review and (6, 7) for meta-analyses].

**Figure 1:**
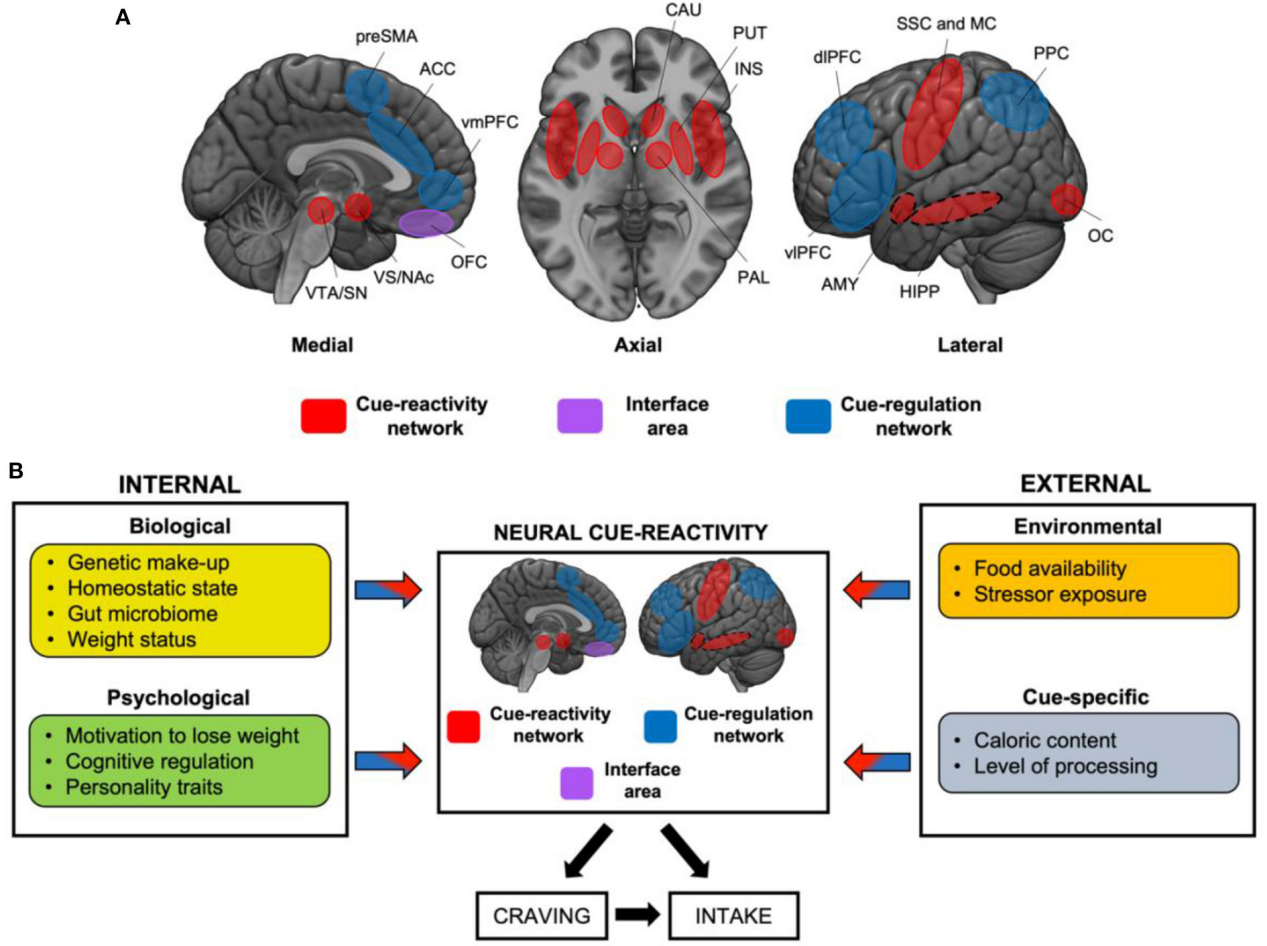
Neural circuits mediating food cue-reactivity: the influence of internal and external factors. **(A)** Cue-reactivity network (red), “interface area” (violet), and “cue-regulation” network (blue). Dashed circles represent medial structures on the lateral surface. OFC may represent an “interface” area, and serve as a target region around which a push-pull balance is reached between the cue-reactivity and the cue-regulation circuits. ACC, anterior cingulate cortex; AMY, amygdala; CAU, caudate; dlPFC, dorsolateral prefrontal cortex; HIPP, hippocampus; INS, insula; MC, motor cortex; NAc, nucleus accumbens; OC, occipital cortex; OFC, orbitofrontal cortex; PAL, pallidum; PPC, Posterior Parietal Cortex; preSMA, pre supplementary motor area; SN, substantia nigra; SSC, somatosensory cortex; VS, ventral striatum; VTA, ventral tegmental area. **(B)** This simplified model, adapted from (10), displays the main internal (biological and psychological) and external factors (environmental and cue-specific) that modulate the neural response to food cues. These factors are expected to act in isolation, by up- or down-regulating the responses of the cue-reactivity and/or cue-regulation network, or in interaction, giving rise to specific brain activation patterns. These, in turn, are expected to influence craving and, ultimately, food intake.

At the functional level, these regions can be categorized into two main circuits: those underlying the sensory and motivational responses (“cue-reactivity”), and those supporting higher-order attentional, decision-making, and inhibitory control processes (“cue-regulation”; Figure 1A). Although the former is more tightly related to “bottom-up” processes prompted by the exposure to cues, and the latter to “top-down” processes, we underline that these two circuits do not always operate separately and, as such, do not represent a dichotomous system. Indeed, they are better represented by a dynamic system in which “interface areas,” such as the orbitofrontal cortex (OFC) (8, 9), may act as a target region where a balance between the circuits is reached in order to orient behavior toward cues or prompting executive control. In other words, similarly to what proposed for substance use disorder, we speculate that the OFC lies at the center of two opposing processes (reward-related go-signals and executive-related no-go signals) (9, 10), therefore acting as a “target” around which the two processes exert their influence over behavior [for a discussion see also (11)].

Recently, Jasinska et al. (12) proposed a model to examine individual-specific (e.g., addiction severity) and study-specific factors (e.g., drug availability) that modulate the neural reactions to drug cues.

Here, we propose a first attempt to translate Jasinska et al.'s model to the domain of food cue-reactivity (Figure 1B): this framework fits well the current literature of eating behaviors (normal and pathological), and it may help identifying transdiagnostic processes that can be targeted by treatment and prevention strategies. Slightly differently from the original model (12), we suggest that the neural response to food cues might be modulated by internal (depending on the status of the organism), and external factors (depending on the environmental and sensory conditions).

We will first describe these internal and external factors, and then address how they might have a role for giving rise to specific brain activation patterns. Finally, we will discuss the importance of our model for framing new research ideas in the domain of food behavior.

## Factors affecting neural food cue-reactivity

### Internal factors: Biological and psychological

Biological factors such as one's genetic make-up can strongly influence the neural reactivity to food cues (both craving and intake). Young adults with the A2/A2 allele (TaqIA rs1800497 polymorphism in chromosome 11) express 30–40% more dopamine D2 receptors (13), a neurotransmitter that is crucial in modulating the motivational value of rewards. This greater availability of dopamine D2 receptors is accompanied by increased activity in the basal ganglia (i.e., the caudate) in response to food cues (i.e., milkshakes), which also predicts greater future weight gain (14, 15) (opposite pattern shown in individuals with the A1 allele). Furthermore, the fluctuating levels of several peripheral homeostatic signals can modulate neural responses to food cues. Orexigenic signals that promote appetitive behaviors, such as the hormone ghrelin, can increase the neural activity of key regions of the cue-reactivity network (i.e., striatum, amygdala and insula) and cue-regulation network (i.e., OFC) in response to visual food cues (16). Conversely, anorexic signals such as insulin (17), leptin (18), or PYY (19) normally dampen such responses.

Of note, recent models on the Brain-Gut-Microbiome axis suggest that a diet rich in fat/sugar and low in fiber is associated with reduced microbial diversity, mucus-stimulating microorganisms, mucus thickness, and increased epithelial leakiness, leading to reduced intestinal barrier function and activation of the gut-associated immune system (20, 21). This state of “metabolic endotoxemia” is thought to reduce central satiety mechanisms by (i) influencing enteroendocrine secretion of satiety hormones such as PYY and cholecystokinin, and by (ii) reducing the expression of anorexigenic peptide receptors on vagal afferents and leptin receptors in the hypothalamus, leading to a disinhibition of satiety mechanisms (22, 23). Despite we are not aware of any study addressing the influence of the gut microbiome on the neural responses to food cues, we believe that this may represent another biological factor worth of investigation.

Growing evidence suggests that another modulating factor is weight status, usually measured with the Body-Mass Index (BMI)^1^. Compared to healthy weight, individuals with obesity show increased activity in areas involved in motivation and habit formation [caudate and nucleus accumbens (NAc)], salience and memory (insula and hippocampus), as well as in regions involved in reward evaluation and goal-directed behaviors (i.e., OFC), while viewing food cues (24–26).

Psychological factors such as the motivation to change one's own dietary habits can shape the neural response to food cues. Compared to ex-dieters, healthy weight individuals, who are currently on diet, show increased activity of regions involved in cognitive control prefrontal cortex (PFC) in response to food cues, suggesting that long-term goals of weight loss can increase the reactivity of the cue-regulation network (27). Interestingly, this difference across dieters was only evident in the fed condition, pointing to higher-level interactions between biological and psychological factors (27). Likewise, the explicit cognitive regulation of craving of foods (e.g., mindful attention, thinking about long-term costs of eating high-calories food) has been associated with increased cognitive control and goal-directed behavior (greater activity in dorsolateral PFC (dlPFC) and OFC) and a concomitant reduced motivation and salience of such cues (decreased activity in ventral tegmental area (VTA), NAc, and amygdala) (28).

Preliminary evidence points to the role of personality traits in this framework. Self-directedness (linked to emotional stability and goal-directed behavior) (29), was negatively associated with emotional regulation (amygdala activity) in response to appetizing food vs. non-foods (30), suggesting that it may represent a protective factor against cue-driven food cravings and intake. Conversely, increased disinhibited eating and trait impulsivity were positively associated with greater insula and amygdala responses to palatable foods (31).

### External factors: Environmental and cue-specific

Environmental factors play an important role in driving the neural reactions to food cues. Compared to the domain of drug addiction (32, 33), fewer studies investigated the effects of food availability on the brain responses. When the food was made available during (or immediately after) cue exposure, healthy weight participants exhibited heightened activity of regions involved in appetitive behaviors, emotional regulation and reward evaluation (striatum, insula, amygdala, and OFC) (34, 35), suggesting the augmented reward value of food when it is readily available for consumption. Exposure to environmental stressors can alter the neural response to food cues and, ultimately, food intake. In women with high self-reported stress, exposure to high-calories food pictures induced greater activity of the striatum, amygdala, and anterior cingulate cortex (ACC) together with a decreased activation of dlPFC compared to low-calorie foods (36), suggesting that high stress may predispose overeating by increasing the motivational value of food and decreasing executive control. Using a guided mental imagery paradigm, overweight/obese women exhibited greater right amygdala activity in response to milkshake intake while imaging a stressful vs. relaxing scenario, and this activity was positively associated with their basal cortisol level (37).

Concerning the actual and perceived caloric content, high-calories vs. low-calories food pictures elicit greater activity of regions involved in motivation and habit formation (dorsal and ventral striatum), salience (amygdala, insula), and reward evaluation (OFC) (25, 38), especially in fasting conditions (38). This heightened reactivity to high-calories food is greater in overweight/obese individuals [see (39) for a meta-analysis]. Recent evidence showed that the level of processing of foods (i.e., raw carrots vs. roasted carrots vs. carrot cake) must be taken into account since this human intervention in modifying the natural state of foods also holds distinct brain representations that are by some means independent from the brain responses to caloric content (40).

## Toward a new model of food cue-reactivity shaping the interplay of internal and external factors

The drive for pleasure is “hard-wired” in our brain (41, 42): from the gratification derived from the fulfillment of biological and social needs, that granted the survival of our species, to the enjoyment of beauty and discovery, which led to the realization of remarkable endeavors in arts and sciences. The relentless search for pleasure guided our evolution. Yet, there are situations where these adaptive motivational processes result in compulsive and addictive-like intake of rewards, whereby highly reinforcing stimuli, such as foods or drugs, disrupt the normal motivational processes and lead to maladaptive behaviors. Frustrating as it might be, greatly pleasurable stimuli such as high-calories food, and drugs of abuse, can “hijack” the same neurocognitive machinery evolved to grant our survival (43, 44).

Here we suggest that the core idea behind Jasinska et al.' model of drug cue-reactivity (12) can be translated in the domain of eating behaviors, unveiling intriguing similarities between the two. We highlighted the role of some major internal and external factors that can influence the neural reactivity to food cues and, ultimately, food craving and intake, suggesting that this grouping of the factors best captures the nature of feeding behaviors as arising from the integration of internal (e.g., homeostatic signals) and external (e.g., food availability) sources of information (45). We argue that Jasinska et al.' claim regarding the importance of the interactive effects of the factors (12) also holds for the domain of food cue-reactivity. Crucially, we propose that the described external and internal factors may act in isolation, by up- or down-regulating the responses of the cue-reactivity/cue-regulation network, or in interaction, giving rise to specific brain activation patterns. These factors are expected to influence craving and, ultimately, food intake. We anticipate that these networks most likely lie in a “dynamic balance:” interface regions such as OFC (8, 10, 11) may serve as a target region around which a push-pull balance is reached between the cue-reactivity and the cue-regulation circuits, as a function of the abovementioned factors. As shown by a recent meta-analysis by Devoto et al. (7), weight status interacts with the homeostatic signals and with the sensory modality of stimulus presentation, reinforcing the notion that individuals with obesity exhibit greater activity in regions involved in motivation (i.e., striatum) in response to visual food cues, despite their satiety state (46). We argue that this impaired central satiety signaling may depend on complex interactions across all levels of the Brain-Gut-Microbiome axis (22, 23): this is made plausible by the observation that obesity is frequently associated with a higher consumption of the kind of food that favors metabolic endotoxemia due to a “bad” gut microbiome. The interaction between food availability and the caloric content of food was also found (35), with higher striatal activity in response to high-calorie (vs. low-calorie) foods only when food was available for immediate consumption.

We believe that there are several reasons why this new perspective may prove useful, for both basic research and translational medicine. First, the evidence that different factors —in isolation and in interaction— can influence the neural response to food cues should lead future studies to acknowledge these effects by controlling for possible confounding variables.

Second, a deeper comprehension of the contextual factors that determine the neural food cue-reactivity and craving is indeed crucial for the development of effective treatments to tackle the current prevalence and rise of obesity (47). Cognitive-Behavioral interventions, particularly if they include the empowerment of cognitive and emotional regulation in response to food cues, may also benefit from the integration of contextual factors into their design. For instance, a Cognitive-Behavioral intervention aimed at reducing food cravings in individuals with obesity may be performed under specific circumstances (e.g., satiety) and with a particular cue (e.g., pictures of high-calorie food). Similarly, brain-centered treatments, whether they involve real-time neurofeedback (48) or the use of non-invasive brain stimulation techniques, were effective in reducing craving and intake for both food (in eating disorders) (49) and drug [in addiction; see (50) for a meta-analysis] and may easily integrate contextual factors into their design.

Dovetailing with this hypothesis, previous studies demonstrated that deep excitatory repetitive Transcranial Magnetic Stimulation (TMS) over the bilateral insula and PFC is effective in inducing weight loss in individuals with obesity (51), and resting-state neuroimaging data suggests that this effect is driven by a decreased reactivity to sensory stimuli, accompanied by an increased reliance on higher-order processes (49). It follows that a fine-grained characterization of the role of contextual factors at the neurofunctional level is essential to develop ecological and personalized treatments.

This fine-grained characterization can only be accomplished by the concomitant manipulation of different internal and external factors: in fact, food cues are usually perceived under specific internal (e.g., homeostatic state) and external contingencies (e.g., food availability; social factors), rather than in the vacuum. With this respect, our opinion paper provides a first pool of factors that can be manipulated by the researcher interested in the brain reaction to food cues. For instance, one might be interested in the differential responses to food cues in healthy weight vs. individuals with obesity, under different homeostatic states (hunger vs. satiety), and when food is available (vs. unavailable).

We acknowledge that the model presented here does not include other factors that can modulate brain responses to food cues, such as sex (52), age (53), sensory modality (7), and length of stimulus presentation. Future studies will help to determine, by modeling internal and external factors in factorial designs, which main effects and interactions are crucial to understand the neurocognitive bases of normal and pathological eating behaviors. As in the domain of drug addiction (12), elucidating such interactions will pave the way to more effective, ecological, and tailor-made (behavioral or brain-centered) interventions.

Finally, we speculate that most factors illustrated here may influence the neural reactivity to different biological (e.g., sexually arousing) and non-biological (e.g., gambling) rewards, in the normal and pathological motivation. We anticipate that multidisciplinary researchers will take up the challenge, enriching our understanding on how the brain copes with pleasurable stimuli in our everyday life.

## Author contributions

FD conceived the original idea of the manuscript. FD and CC wrote the manuscript. FD, CC, EP, and LZ reviewed and finalized the manuscript. All authors contributed to the article and approved the submitted version.

## Funding

This manuscript was supported by a grant funded by the Italian Ministry of Health (Ricerca Corrente; Project L4119; PI: LZ).

## Conflict of interest

The authors declare that the research was conducted in the absence of any commercial or financial relationships that could be construed as a potential conflict of interest.

## Publisher's note

All claims expressed in this article are solely those of the authors and do not necessarily represent those of their affiliated organizations, or those of the publisher, the editors and the reviewers. Any product that may be evaluated in this article, or claim that may be made by its manufacturer, is not guaranteed or endorsed by the publisher.

## Footnotes

^1^The BMI is an indirect measure of adiposity and obesity severity. It is calculated as weight (in kilograms)/height squared (in meters). Healthy weight: 18.5 BMI < 25; underweight: BMI < 18.5; overweight: 25 BMI < 30; obese: BMI 30.
